# Deconvolution of Adult T-Cell Leukemia/Lymphoma With Single-Cell RNA-Seq Using Frozen Archived Skin Tissue Reveals New Subset of Cancer-Associated Fibroblast

**DOI:** 10.3389/fimmu.2022.856363

**Published:** 2022-04-07

**Authors:** Eun-Hye Joo, Jai Hee Bae, Jihye Park, Yoon Ji Bang, Joseph Han, Nicholas Gulati, Jong-Il Kim, Chung-Gyu Park, Woong-Yang Park, Hyun Je Kim

**Affiliations:** ^1^Samsung Genomic Institute, Samsung Medical Center, Seoul, South Korea; ^2^Samsung Advanced Institute of Health Science and Technology, Sungkyunkwan University, Seoul, South Korea; ^3^Department of Dermatology, Samsung Medical Center, Seoul, South Korea; ^4^Department of Biomedical Science, Seoul National University Graduate School, Seoul, South Korea; ^5^Department of Dermatology, Icahn School of Medicine at Mount Sinai, New York, NY, United States; ^6^Genome Medicine Institute, Seoul National University College of Medicine, Seoul, South Korea; ^7^Cancer Research Institute, Seoul National University College of Medicine, Seoul, South Korea

**Keywords:** adult T-cell leukemia/lymphoma, single-cell RNA-seq, cancer-associated fibroblast, frozen tissue, epidermal growth factor receptor pathway

## Abstract

Adult T-cell Leukemia/Lymphoma (ATLL) is a rare aggressive T-cell malignancy caused by human T-cell leukemia virus type 1 (HTLV-1) infection. However, little is known about the underlying activated molecular pathways at the single cell level. Moreover, the intercellular communications between the tumor microenvironment (TME) and tumor cells in this malignancy are currently unknown. Difficulties in harvesting fresh tissue in a clinical setting have hampered our deeper understanding of this malignancy. Herein, we examined ATLL using archived fresh frozen tissue after biopsy using single-cell RNA sequencing (scRNA-seq) with T-cell receptor (TCR) clonal analysis. Highly clonal tumor cells showed multiple activating pathways, suggesting dynamic evolution of the malignancy. By dissecting diverse cell types comprising the TME, we identified a novel subset of cancer-associated fibroblast, which showed enriched epidermal growth factor receptor (EGFR)-related transcripts including early growth response 1 and 2 (EGR1 and EGR2). Cancer associated fibroblasts (CAFs) of ATLL play an important role for CD4 T-cell proliferation *via* FGF7-FGF1 and PDGFA-PDGFRA/B signaling, and CAFs, particularly EGR-enriched, are also associated with CD8 and NKT expansion by EGFR. These findings suggest a potential targeted therapeutic pathway to better treat this neoplasm.

## Introduction

Adult T-cell Leukemia/Lymphoma (ATLL) is an aggressive mature T-cell neoplasm caused by human T-cell leukemia virus type 1 (HTLV-1) infection (1, 2). Since HTLV-1 infection is endemic in southwestern Japan, ATLL has been mainly reported in the same region (3, 4). Recent advances in next-generation sequencing (NGS) technology have provided more detailed information on the unique pathogenesis of ATLL compared to other subtypes of peripheral T-cell lymphoma (PTCL) (5–7). Copy number abnormalities (CNAs) of ATLL are comparable to the PTCL-GATA3 subgroup (5), and gene expression profiling has been used to define distinct diagnostic and prognostic subtypes of PTCL (8). However, investigations using single-cell RNA sequencing (scRNA-seq) technology with T-cell receptor (TCR) clonal analysis have been lacking, making it difficult to understand the dynamics of the immune response during ATLL progression. HTLV-1 not only infects T-cells, but also various cell types including B-cells, myeloid cells, and fibroblasts (9, 10). Accordingly, the interaction with the tumor microenvironment (TME) surrounding the neoplasm is important for its regulation and growth. ScRNA-seq has provided a useful cell atlas to better understand the intra-tumoral diversity of the TME and disease-specific cellular crosstalk with malignant cells (11). In this study, we used scRNA-seq and TCR clonal analysis to dissect the malignant tumor cells and TME of ATLL. With this approach, we were able to identify distinct T-cell subpopulations that likely represent the malignant clones in ATLL *via* an integrated analysis of the transcriptome and T-cell clonal repertoire. We also examined non T-cell components of the ATLL TME including myeloid cells and stromal cells, especially cancer associated fibroblast (CAF) subtypes and their potential communications with T-cells. In this study, we sought to identify a new possible target for ATLL treatment, while also considering clonal malignancy and their TME interactions.

## 2 Methods

### Patient Information

Human skin samples were obtained using remnants of biopsy tissue taken for diagnostic purposes under an Institutional Review Board (IRB)-approved protocol (IRB# 2020-03-060). For this study, we used a fresh frozen tissue sample from a 69-year-old man who presented to our clinic with newly developed erythematous nodules of his bilateral axillae, inguinal areas, and flexural surfaces of the arms. At the time of tissue profiling, he had not been diagnosed nor underwent any treatment. The samples were obtained by 4 mm skin punch biopsies of the right axilla. In laboratory investigations, lactate dehydrogenase (LDH) was elevated to 392 U/L (normal range <225 U/L) and B2-microglobulin was elevated to 3.47 μg/mL (normal range < 2.4 μg/mL).

### Tissue Collection and Dissociation

Specimens were placed in phosphate-buffered saline (PBS) on ice. Biopsy samples were cryopreserved in optimal cutting temperature (OCT) compound and stored at -80°C. For cell dissociation, cryopreserved tissue was thawed in a 37°C water bath and transferred to freshly prepared dissociation solution composed of 200 μL of Liberase TL (2 mg/mL; Sigma Aldrich) and 1800 μL PBS, and incubated at 37°C for 15 min. The tissue was manually disaggregated, using a 1 mL pipette with a wide bore and gently pulling the solution up and down 10 times. The cells were collected through a 70-μm cell strainer (#352340, Corning) and stored on ice. The tissue was transferred to a dissociation solution for a second round of dissociation as noted above, followed by dissociation in Trypsin solution (350 μL PBS, 50 μL 0.25% Trypsin). Cells were washed once and re-suspended in 100 μL of freshly prepared PBS-bovine serum albumin (BSA; 1 x PBS and 0.04% BSA) and processed on the 10x Genomics platform.

### Library Construction for Single Cell Gene Expression and TCR Profiling and NGS Sequencing

Single cell dissociates were loaded into the Chromium system (10x Genomics, USA) to encapsulate into a single droplet targeting approximately 25,000 cells. The Chromium Single Cell 5’ Kit (10x Genomics, USA) was used to generate scRNA-seq and TCR libraries, according to the manufacturer’s instructions. Briefly, single Cell 5’ Kit enables the measurement of gene expression and the immune repertoire from the same cells, profiling the full-length of 5’ UTR and paired TCR transcripts from individual cells. Chromium Controller™ splits the cells into nano-scale Gel Beads-in-emulsion (GEM), where barcoded cDNA was generated. The TCR library was constructed by PCR amplification of GEM with TCR region specific primers, whereas the gene expression library was made without V(D)J segment amplification. Each library was loaded on a NovaSeq 6000 platform (Illumina, USA) with pair-end reads of 150 bp to generate the sequencing data.

### Data Processing

ScRNA-seq and TCR-seq data were pre-processed and aligned to the human reference genome (GRCh38) using the CellRanger 4.0.0 pipeline (https://support.10xgenomics.com/single-cell-vdj/software/pipelines/latest/what-is-cell-ranger). Raw sequence base call (BCL) files were converted into FASTQ files using the “mkfastq” command. For scRNA-seq, the “count” command was used to align the reads to the genome, annotate with transcripts, and count UMI with correction steps. For TCR-seq, the “vdj” command was used to assemble the reads into contigs and annotated with V, D and J segments and CDR3 regions. Gene expression matrix from the CellRanger count was filtered, normalized using the Seurat 3.1.4 in R 4.0.5 software (R Foundation for Statistical Computing, Vienna, Austria) and selected according to the following criteria: cells with >200 genes; and <20% of mitochondrial gene expression in unique molecular identifier (UMI) counts. We used “filtered_contig_annotations” determined by Cell Ranger vdj, which contained the alpha chain, beta chain and CDR3 nucleotide sequences by each barcode. Following QC, scRNA-seq and TCR-seq data were merged on the Seurat objects.

### Dimensionality Reduction, Clustering, and Differential Expression Analysis

Dimensionality reduction and clustering were done as recommended by the Seurat developers (12). Briefly, gene expression counts were LogNormalized, 2,000 variable features were selected and scaled to the expression level for Principal Components Analysis (PCA). PCA allowed reduction of the high variable gene expression data set into a low-dimensional space for characterizing transcriptional profiles (13). Clustering was performed using louvain algorithm based on PCs, and Uniform Manifold Approximation and Projection (UMAP) was used to visualize clustering results into two dimensions (14). Each cluster was manually annotated with canonical cell type features. Differentially expressed genes across the clusters were performed using Model-based Analysis of Single-cell Transcriptomics (MAST) test (15).

### Copy Number Variation Analysis

To identify malignant tumor cells with chromosomal copy number changes, we used inferCNV 1.6.0 (https://github.com/broadinstitute/inferCNV). The raw gene expression data were extracted from the Seurat object and public single-cell data derived from a healthy donor (16) were included as a normal control reference.

### TCR Repertoire Analysis

We analyzed outputs of CellRanger vdj (10x Genomics) that included the CDR3 sequences and clonotype of assembled alpha and beta chains of TCR. We also analyzed the V-gene usage and the frequency of both alpha and beta chains to investigate the variation across the clonotype. Weblogo plots were generated using WebLogo 3.7.4 (https://weblogo.berkeley.edu). Sequence logos are a graphical representation for conservation of amino acid sequence alignment and height of the symbol indicates the relative frequency of each amino at that position.

### Cell-Cell Interaction Analysis

To investigate the potential cell-cell communication between ATLL T-cells and other cell types including stromal cells, we applied CellChat 1.1.2 (https://github.com/sqjin/CellChat) (17) with scRNA-seq data. CellChat inferred the potential cell-cell ligand-receptor interaction between assigned cell types and visualized into a diagram.

### Resource Availability - Data and Code Availability

10x Genomics peripheral blood mononuclear cells (PBMCs) datasets from a healthy donor are available at the 10x Genomics website (16).

## Results

### Patient Characteristics

**Figure 1:**
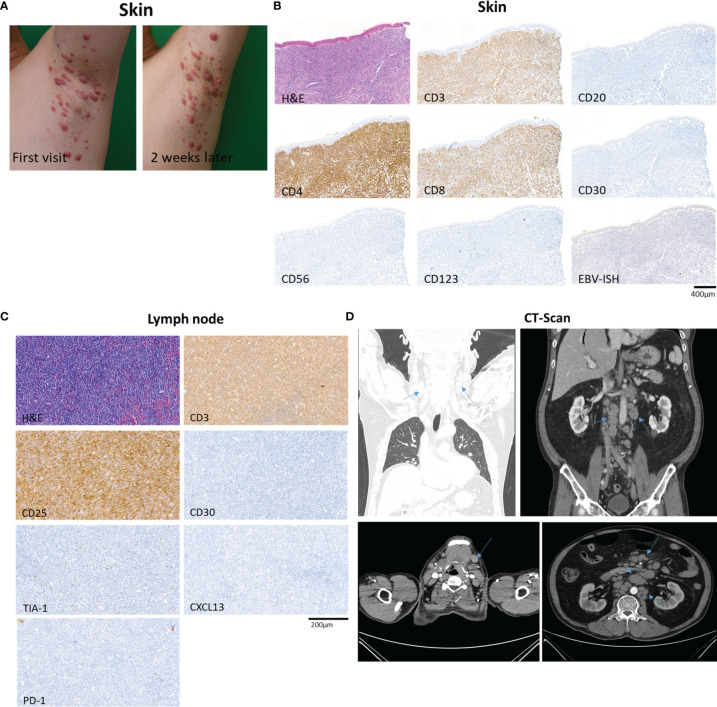
Overview of a patient with adult T-cell leukemia/lymphoma involving skin and lymph node. **(A)** The patient’s skin lesions at first visit and 2 weeks after the first visit, showing rapidly progressing erythematous papules and nodules of the left axilla. **(B)** Biopsy specimen of skin showing dense infiltration of atypical lymphocytes with predominance of CD4+ over CD8+ T cells, and negative expression of CD30, CD20, CD56, and CD123. In situ hybridization of EBV was negative. **(C)** Excisional biopsy specimen of neck lymph node demonstrating CD4+ and CD25+ non-cytotoxic mature T-cell lymphoma involvement. **(D)** Computed tomography of chest, abdomen and pelvis showing abnormal lymph node enlargement (arrows) in the abdomen, pelvis, and bilateral neck, highly suspicious of lymphoma involvement.

A 69-year-old man with a history of hypertension presented with multiple erythematous papules and nodules of the bilateral axillae, inguinal areas, and flexural surfaces of the arms. At the 2-week follow-up visit, the skin lesions had increased both in number and size (**Figure 1A**). A biopsy of a palpable mass of the neck favored T-cell lymphoma. Chest and abdominal computed tomography showed bilateral neck as well as abdominal and pelvic lymph node enlargement, suspicious for lymphoma involvement (**Figure 1D**). Diagnostic bilateral bone marrow biopsies were performed, and no lymphoma was found. Positron emission tomography/computed tomography done for staging was consistent with stage III lymphoma. Skin biopsy showed dense infiltration of atypical lymphocytes. Immunohistochemical studies demonstrated predominance of CD4+ over CD8+ T cells, and CD30, CD20, CD56, CD123 were all negative in the lymphocytic infiltrate (**Figure 1B**). Lymphocytic component stained positive for Ki-67 in 95% of tumor cells. The patient was initially diagnosed with peripheral T-cell lymphoma, not otherwise specified. After discussion with the hematology/oncology team, the patient was started on combination chemotherapy consisting of cyclophosphamide, doxorubicin, vincristine, and prednisone. After six courses of chemotherapy, the patient was free of new skin lesion. The patient’s biopsy specimen of a neck lymph node showed CD4+ and CD25+ non-cytotoxic mature T-cell lymphoma (**Figure 1C**). Due to suspicion for ATLL, HTLV-1 polymerase chain reaction (PCR) was done, and the result was positive. Thus, the patient ultimately received a diagnosis of ATLL.

### Annotation of the Multiple Cell Types Comprising ATLL Skin by scRNA-Seq

**Figure 2:**
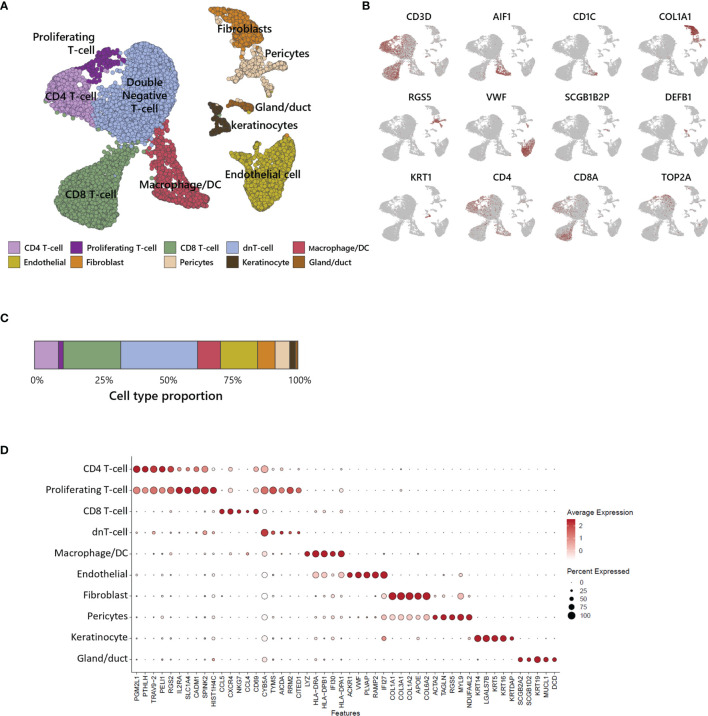
Single-cell transcriptomic analysis of ATLL. **(A)** UMAP plot for 15,550 skin cells clustered by unsupervised Seurat clustering and annotated with 10 cell types. **(B)** Each cluster is identified by canonical cell type marker expression. **(C)** Proportion of each cell type in ATLL skin sample. **(D)** Dot-plot showing scaled average gene expression of the top 5 differentially expressed genes in each cluster of **(B)**.

We performed scRNA-seq analysis to better investigate the cellular composition of ATLL (**Figure 2A**). After filtering-out for quality assessment, a total of 15,550 cells were obtained. Unbiased clustering followed by UMAP dimension reduction revealed 10 distinct cell clusters according to gene expression pattern (**Figure 2D**). Each cluster was well characterized by the transcriptional profile representing specific cell types. We annotated cell type identity in each cluster with highly expressed canonical markers (**Figure 2B**): CD3D for T-cells, ALF1 and CD1C for macrophages and dendritic cells (DCs), COL1A1 for fibroblasts, RGS5 for pericytes, VWF for endothelial cells, SCGB1B2P for eccrine gland/duct cells, and DEFB1 and KRT1 for keratinocytes. Among CD3D^+^ T-cells, we found the following subtypes according to surface markers: CD4^-^/CD8^-^ T-cells (double negative, dnT), CD4^+^ T-cells, proliferating CD4^+^ T-cells with TOP2A, and CD8^+^ T-cells. We found that 62% of ATLL cells were T-cells (**Figure 2C**), suggesting significant expansion of malignant T-cells as a characteristic of ATLL. Other cell type proportions were as follows: 9% for macrophages/DCs, 7% for fibroblasts, 14% for endothelial cells, 5% for pericytes, 2% for keratinocytes, and 1% for gland cells.

### Heterogeneity of T-Cells and Tumor Identification Within ATLL

**Figure 3:**
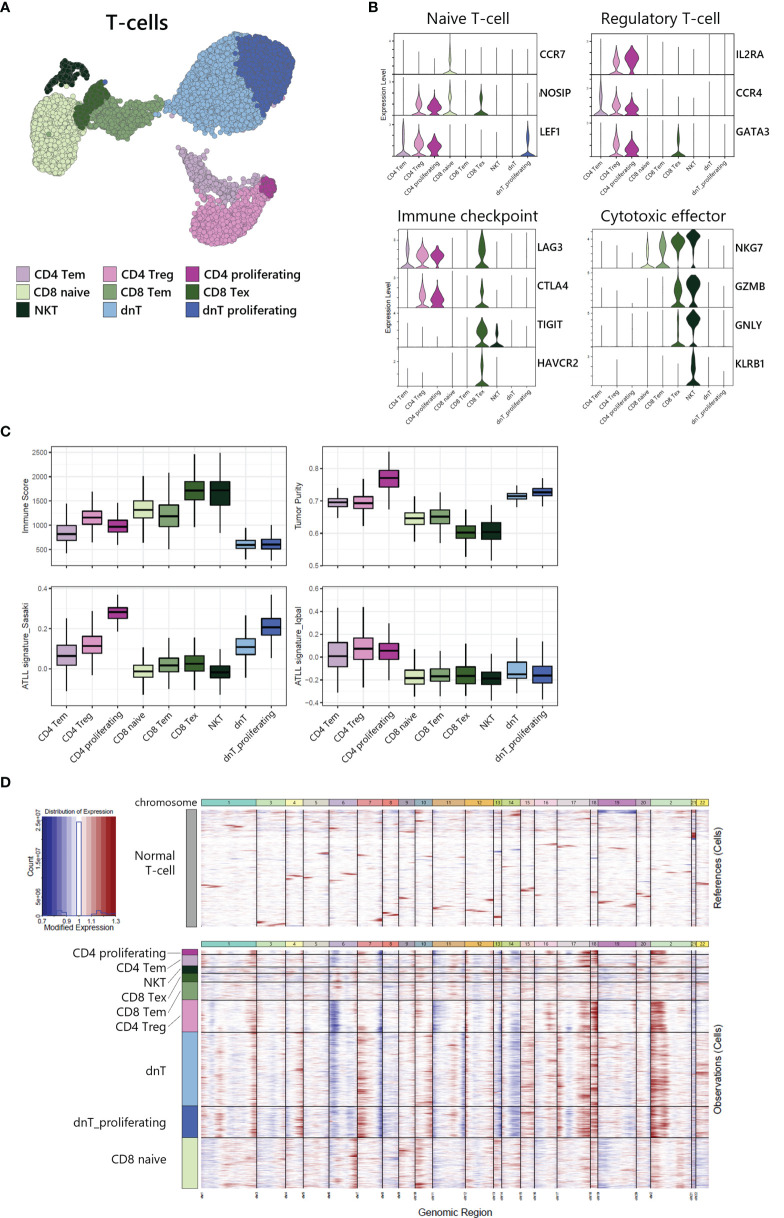
Tumor identification within T-cell subpopulation. **(A)** UMAP of the 9,625 cell T-cell population reveals 9 distinct clusters. **(B)** Functional gene expression of T-cell subtypes. **(C)** Estimated Immune Score and Tumor Purity of T-cell subtypes. **(D)** Heatmap of inferred copy number changes (infercnv) compared to T-cells from healthy donors.

To investigate the heterogeneity within T-cells, we sub-analyzed 9,625 T-cells and revealed 9 sub-clusters (**Figure 3A**). Each sub-cluster was annotated based on relative expression of functional genes related to immune status (**Figure 3B**). CD4+ T-cells were separated into 3 sub-populations: CD4 effector memory T-cells (Tem) expressing MAL and RORA (18) (**Supplementary Figure S1**), CD4 regulatory T-cells (Treg) expressing IL2RA, CCR4 and GATA3, and proliferating T-cells expressing MKI67 and TOP2A (**Supplementary Figure S1**). CD8 T-cells were separated into 3 sub-populations: CD8 naive T-cells expressing CCR7 and NOSIP with less expression of effector genes compared to other CD8 T-cells, CD8 Tem expressing cytotoxic effectors such as NKG7, GZMA, and GZMK, and CD8 exhausted T-cells expressing immune checkpoint genes such as LAG3, CTLA4, TIGIT, and HAVCR2. NKT was identified by CD3D, NKG7, GNLY, and KLRB1 expression. dnT-cells rarely expressed functional genes, whereas dnT proliferating cells mainly expressed cell-cycle related genes such as STMN1, TOP2A, UBE2C, and MKI67 (**Supplementary Figure S1**).

To differentiate the tumor and non-tumor immune cells, we utilized the ESTIMATE (19) algorithm to compare predicted immune proportion and tumor purity across T-cell subtypes (**Figure 3C**, upper). CD4+ cells (CD4 Tem, CD4 Treg, and CD4 proliferating) and dnT-cells (dnT and dnT proliferating) showed lower immune score but higher tumor purity compared to CD8 T-cells and NKT. Further, we computed module scores using previously suggested ATLL signatures by Sasaki (20) and Iqbal (8) (**Figure 3C**, bottom). All three types of CD4+ T-cells and all dnT-cell types exhibited marked scores for ATLL signatures, but CD8+ T-cell types and NKT scored low, suggesting tumor malignancy of ATLL is more associated with CD4+ and dnT-cells than CD8+ and NKT cells.

Next, to evaluate the genomic variation within T-cells, we used inferCNV to identify the copy number variation (CNV) between each cell subtype with T-cells from a healthy donor as a normal control (**Figure 3D**). CD4+ T-cells and dnT-cells were found to have multiple chromosomal changes (gain of 1q, 2p, 7p, 17q, 18p and 18q, and loss of 6p, 7q, 13q, and 14q) compared to CD8+ T-cells, which suggests that CD4+ T-cells accumulated more CNV abnormalities compared to normal T-cells. In a previous genomic study, Heavican observed the same pattern of CNV aberration mainly in GATA3 T-cell lymphoma (5). Consequently, CD4+ T-cells and dnT-cells can be defined as a malignant tumor within ATLL, based on transcriptomic characteristics and either type of CNV abnormalities.

### Single-Cell V(D)J Recombination Repertoire Analysis of T-Cell Receptors

**Figure 4:**
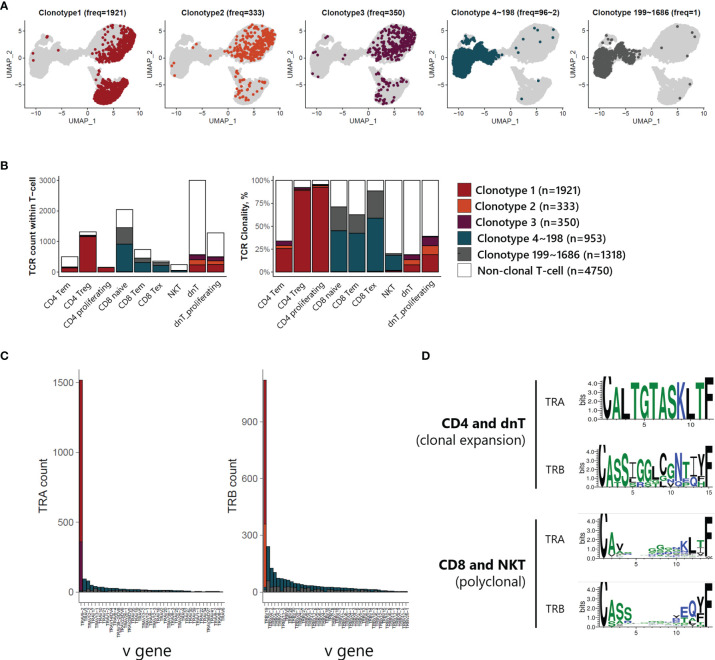
TCR analysis of T-cells of ATLL **(A)** TCR clonotype expansion within T-cells **(B)** Proportional expansion of each TCR clonotype within the different T-cell subtypes **(C)** Composition of variable gene of TRA and TRB **(D)** Weblogo plot showing conserved amino acid sequence of TRA and TRB according to T-cell subtypes.

V(D)J recombination sequencing revealed clonally expanded T-cell subtypes with scRNA-seq. We identified 3 largely expanded clones (clonotypes 1, 2, and 3), which accounted for 53% of TCR-expanded cells, and other polyclonal clones (clonotypes 4~1686) by overlapping each clonotype onto UMAP plot of T-cells (**Figure 4A**). Largely expanded clones were mainly detected in CD4 Treg and proliferating T-cell populations, while polyclonal T-cells were observed in CD8 naive, CD8 Tem, and CD8 Tex populations (**Figure 4B**), suggesting that malignancy is originating in the CD4 T-cell compartment. Further, variable gene (V gene) usage in T-cell receptor alpha and beta (TRA and TRB) showed that largely expanded clones had uniform repertoires using the same V gene, TRAV9-2 for TRA of clonotypes 1 and 3, and TRBV19 for TRB of clonotypes 1 and 2 (**Figure 4C**). We also analyzed the CDR3 amino acid sequence conservation of TRA and TRB according to TCR clonality (**Figure 4D**). In the case of TCRs in CD4 T-cells and dnT-cells, sequence prevalence was strongly conserved to CALTGTASKLTF for TRA and CASSIGGLCGNTIYF for TRB. In the case of TCRs in CD8 T-cells and NKT, there was no representative sequences due to the variation in the middle of the sequence (5 to 10).

### Gene Expression Profiles of Clonally Expanded T-Cells and Polyclonal T-Cells in ATLL

To investigate the transcriptomic differences between clonally expanded T-cells and polyclonal T-cells, we analyzed differentially expressed genes (DEGs) in ATLL T-cells compared to healthy T-cells (**Supplementary Figure S2**). We discovered 117 genes up-regulated in T-cells from ATLL compared to T-cells from a healthy donor (**Supplementary Figure S2A**). Up-regulated genes in ATLL were related to metabolic pathways, phosphorylation, cytokine production, cell differentiation, and growth factor stimuli, suggesting that ATLL is a metabolically active cancer type. Down-regulated genes in ATLL were mainly related to lymphocyte activation, immune system development, and hematopoiesis, suggesting the loss of normal lymphocytic differentiation (**Supplementary Figure S2B**). Among 117 ATLL-specific DEGs, we identified 61 genes that were up-regulated in the clonally expanded T-cell group, and 26 in the polyclonal T-cell group (**Table 1** and **Supplementary Figure S2C**). Clonally expanded T-cells had up-regulation of genes related to metabolism (ENO1 and PKM), immunity (CADM1), differentiation (CITED1 and TCF4), oxidoreductase (PRDX1 and TECR) and ATLL pathways (CAV1, CD99 and PTHLH). In contrast, polyclonal T-cells had up-regulation of genes related to HTLV-1 infection (NFKBIA and EGR1), cytokine interaction (CCL4 and IL2RG), apoptosis, and inflammatory response, as well as genes down-regulated in angioimmunoblastic T-cell lymphoma (AILT). Our finding of polyclonal T cells, and not clonal T cells, showing a HTLV-1 infection signature suggests the role of HTLV-1 infection in mediating the initial process of malignancy rather than clonal expansion.

**Table 1:** Differentially expressed genes in T-cells of ATLL compared to healthy donor.

Clonality	Number of unique genes	Functional category	Gene symbols
CD4 and dn T-cell (clonally expanded)	61	Metabolic	NDUFV2, NME1, NME2, ADA, COX5A, ENO1, GAPDH, HPGDS, PKM, RRM2, TYMS, TPI1
		HTLV-1 infection	RAN, SLC25A5
		Immunity	CADM1, HMGB1, HMGB2, ISG20, MIF, PTMS
		Differentiation	CITED1, NME1, CADM1, GTSF1, STMN1, TCF4
		Oxido reductase	NDUFV2, COX5A, GAPDH, PRDX1, PRDX3, RRM2, TECR
		ATLL-related	TYMS, TUBB, UBE2C, NME1, PRDX1, CD99, HMGB2, SLC25A46, ISG20, HPGDS, CAV1, CADM1, PTHLH
CD8 and NKT (polyclonal)	26	HTLV-1 infection	FOS, NFKBIA, ZFP36, CREM, EGR1, IL2RG
		Cytokine-cytokine receptor interaction	CCL4, CXCR4, IL2RG
		Apoptosis	ARL6IP1, TNFAIP3, PPP1R15A, SRGN
		Inflammatory response	CCL4, TNFAIP3, ANXA1
		Down-regulated genes in angioimmunoblastic T-cell lymphoma (AILT)	PPP1R15A, TNFAIP3, ZFP36, FOSB, NR4A2, TSC22D3, RGCC, CREM, YPEL5

### Heterogeneity of Myeloid Cells in ATLL and Characterization of Tumor-Associated Macrophages (TAMs)

**Figure 5:**
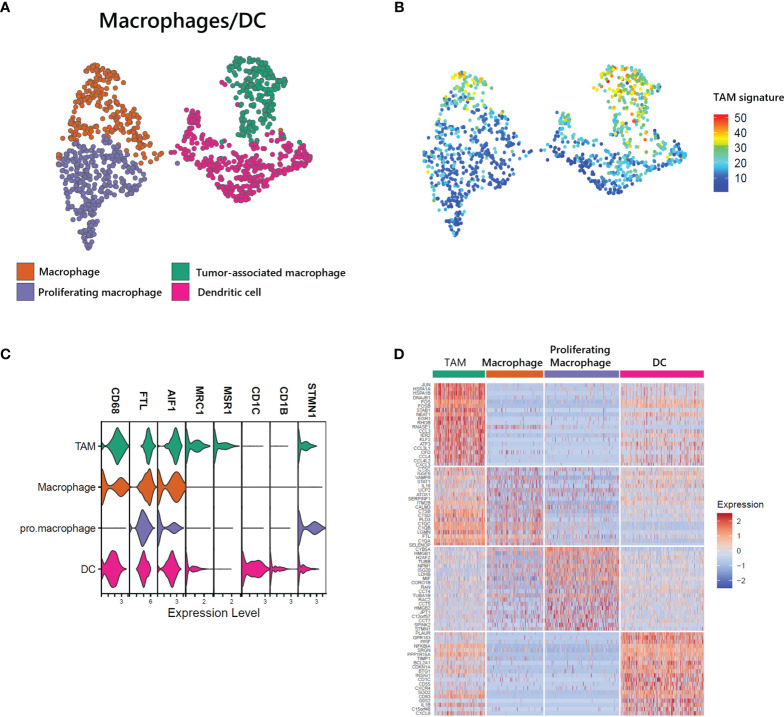
Identification of tumor-associated macrophages (TAMs) in ATLL **(A)** Subtypes of myeloid cells **(B)** TAM score calculated with the gene set suggested by Bagaev (17) within myeloid cells **(C)** Cell-type markers of myeloid cells **(D)** Top DEGs of myeloid cell subtypes.

Myeloid cells, including macrophages and DCs, are closely associated with survival in T-cell lymphoma patients, and the presence of TAMs has been used as a predictive biomarker (21). We found a large proportion of myeloid cells in our ATLL sample (1,181 of 15,550 total cells) and identified 4 subtypes (**Figure 5A, C**): macrophages with FTL, proliferating macrophages with STMN1, TAMs with MRC1 and MSR1, and DCs with CD1C. Further, the computed TAM signature suggested by Bagaev (22) was dominant in the TAM cluster of ATLL (**Figure 5B**). TAMs of ATLL showed a distinct tumor-associated gene expression signature, sharing innate immunity-associated genes (C1QA, C1QB, and C1QC) with the macrophage cluster (**Figure 5D**). Proliferating macrophages exhibited depleted innate immunity gene expression. Instead, NPM1, which is involved in non-Hodgkin lymphoma and acute myelogenous leukemia (23), and CCTs (CCT4, CCT5, and CCT7) were up-regulated. DCs of ATLL showed a similar transcriptional pattern to that of TAMs, and their correlation was relatively high (**Figure 5D** and **Supplementary Figure S3A**). The high expression of CD1C, CD14 and FCER1 suggests that they are closely related to monocyte-derived DCs (moDCs) (24). The expression of several cytokine genes (CD40, CXCR4 and IL1RN) in DCs characterizes inflammatory DCs (iDCs) as well (**Supplementary Figure S3B**). We observed that some of the TAMs and DCs of ATLL exhibited inflammatory properties, with moDCs sharing similar characteristics to TAMs.

### Heterogeneity of Stromal Cells in ATLL and Characterization of CAFs

**Figure 6:**
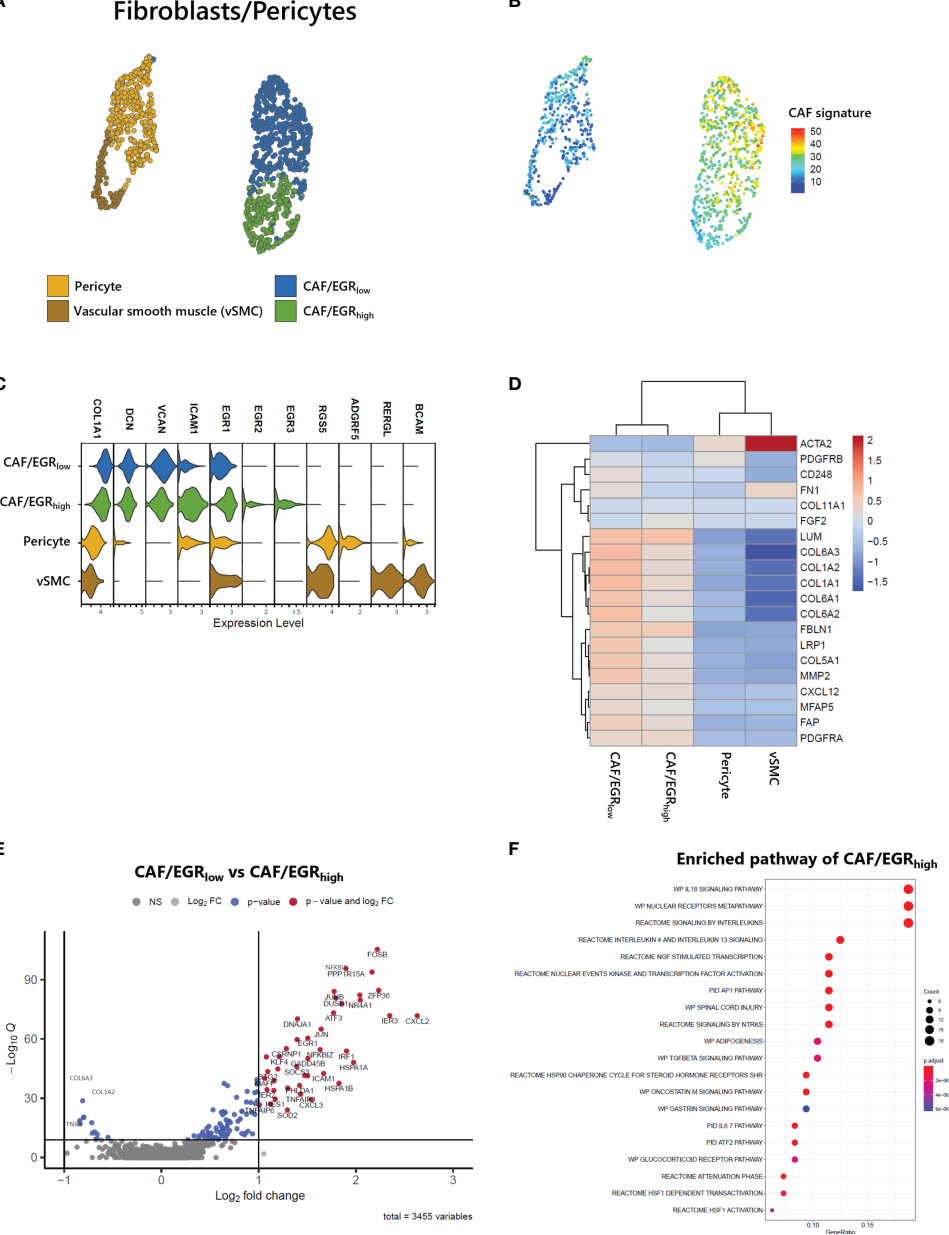
Identification of CAFs in ATLL **(A)** Subtypes of stromal cells **(B)** CAF score calculated with the gene set suggested by Bagaev (17) within stromal cells **(C)** Cell-type markers of stromal cells **(D)** Scaled gene expression related to CAFs **(E)** Volcano plot showing DEGs of CAF subgroups **(F)** Enriched pathway of CAF/EGR_high_ subtype.

Stromal cells, including fibroblasts and pericytes, play an important role in cancer initiation and progression. Pericyte-fibroblast transition has often been associated with tumor invasion and metastasis (12). We analyzed 1,212 stromal cells and identified two vascular cell subtypes, pericytes and vascular smooth muscle cells (vSMC) and two CAF subgroups (**Figure 6A**). While all stromal cell types expressed COL1A1, only fibroblast cell types expressed DCN, and only vascular cell types expressed RGS5 (**Figure 6C**). Both fibroblast cell types exhibited a CAF signature (22) as seen in the UMAP of stromal cells (**Figure 6B**). Within the CAF-related gene set, ACTA2, PDGFRB, and FN1 were not associated with CAFs of ATLL (**Figure 6D**). Rather, LUM, FBLN1, LRP1, COL5A1, MMP2, FAP, and PDGFRA were strongly expressed only in CAFs of ATLL. Since MMP2 is important for extracellular matrix digestion, we speculate that tumor cells promote fibroblast to secret matrix digestion products to facilitate metastasis. However, the cellular mechanism for how the cancer cells regulate the gene expression pattern of other cell types merits further investigation.

CAFs of ATLL were separated into two subgroups: CAF/EGR_high_ exhibited relatively higher expression of epidermal growth response (EGR) genes such as EGR1, EGR2, EGR3, and ICAM1, while CAF/EGR_low_ showed relatively lower expression of these genes (**Figure 6C**). The CAF/EGR_low_ subgroup had relatively increased expression of CAF-related genes compared to the CAF/EGR_high_ subgroup (**Figures 6B, D**), suggesting that the CAF/EGR_low_ subgroup is the primary contributor to tumorigenesis among the two CAF subgroups. Next, we analyzed DEGs between the two CAF subgroups to characterize their functional differences. Forty-two genes were up-regulated in the CAF/EGR_high_ subgroup, but none exceeded the cut-off in the CAF/EGR_low_ subgroup (**Figure 6E**). We performed an enriched signaling pathways analysis using the clusterProfiler package (V3.18.1) (25) using up-regulated genes in CAF/EGR_high_. CAF/EGR_high_ was considered highly related to interleukin signaling such as IL18, IL4, IL13, and IL6 (**Figure 6F**), suggesting that the CAF/EGR_high_ subgroup plays a key role in inflammatory responses, cytokine induction, and proliferation of fibroblasts.

### Cell-Cell Interactions Between T-Cells and CAFs in ATLL Mediated by Growth Factors

**Figure 7:**
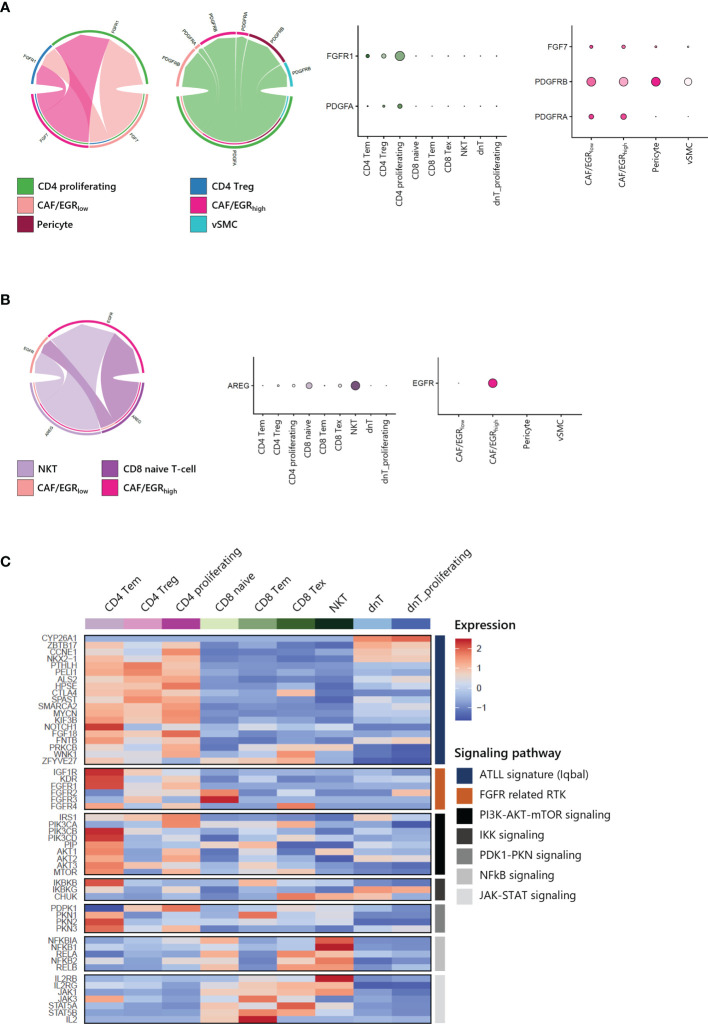
Cell-cell interactions within ATLL **(A)** FGF and PDGF signaling between CD4 T-cells and CAFs **(B)** EGF and AREG signaling between CD8 T-cells and CAF/EGR_high_ **(C)** Signaling pathways in T-cell subpopulations.

To gain further insight into the potential interaction between T-cells and CAFs of ATLL, we analyzed cell-cell interaction acting through ligands and their suggested receptors. By analyzing the types and expression level of known membrane-bound factors in each cell type, we can infer signaling crosstalk between the cell types (17). We found that proliferating CD4 T-cell and CD4 Tregs abundantly expressed FGFR1 as a receptor for FGF7 from stromal cells (**Figure 7A**, left). Additionally, proliferating CD4 T-cells strongly expressed PDGFA as a cognate ligand of PDGFRA and PDGFRB, which were abundantly expressed on CAFs and pericytes (**Figure 7A**, right). For NKT and CD8 naive T-cells, there was abundant expression of AREG (amphiregulin), and CAF/EGR_high_ exclusively expressed EGFR as a cognate receptor of AREG (**Figure 7B**). Potential communications between T-cells and CAFs suggest that CD4 T-cell expansion is closely affected by CAF activity mediated by FGFR1. Moreover, both CD4 T-cells and CD8 T-cells contribute to CAF development through distinct signaling pathways.

Further, we focused on the canonical genes involved in FGFR signaling and its downstream pathways such as PI3K-AKT-mTOR, IKK, PDK1-PKN, NFkB and JAK-STAT signaling in different T-cell subtypes (**Figure 7C**). ATLL-related genes were up-regulated in both CD4 T-cells dnT-cells, but receptor tyrosine kinases (RTKs) such as IGF1R, KDR (VEGFR), and FGFR1 were mainly expressed in CD4 T-cells. Also, RTKs demonstrated a potential network with up-regulated genes of clonally expanded T-cells in **Table 1** (**Supplementary Figure S4**). PI3K-AKT-mTOR signaling genes, which are frequently up-regulated in lymphomas including ATLL, were primarily up-regulated in CD4 Tem and CD4 proliferating T-cells. However, NFkB and JAK-STAT signaling genes were up-regulated in CD8 T-cells and NKT. Since ATLL is thought to originate primarily from CD4 T cells, we posit that IGF1R, KDR (VEGFR), and FGFR1 may serve as potential therapeutic targets for future treatments of ATLL.

## Discussion

In this study, we propose new therapeutic targets for this rare, aggressive malignancy using clinically feasible sample archiving, processing, profiling, and analysis pipelines. Indeed, majority of investigators using RNAseq consider fresh frozen tissue to not be suitable for single ‘cell’ gene expression profiling with TCR analysis given the technical difficulty of the experiments. However, we successfully profiled more than 10,000 cells from metabolically active skin tumors that were vulnerable to tissue processing with the 10X Genomics platform.

ATLL is typically characterized by proliferation of CD4+ and CD25+ T-cells since HTLV-1 mainly infects CD4+ T cells and induces proliferation of this cell subset (1). Clonal proliferation contributes to increasing the number of HTLV-1-infected cells and thus development of ATLL (26), and a recent study demonstrated a strong correlation between the clonality pattern and tumor progression (27). In the patient studied herein, we observed the malignant clonal expansion of CD4+ cells.

The molecular features of ATLL are mostly induced by HTLV-1 infection (28). HTLV-1 induced Th2/Treg-related chemokine receptor CCR4 is frequently expressed in ATLL (2, 29). Moreover, CCR4 is known as a GATA3 target gene that is responsible for FOXP3 expression and controlling Treg function (30). In malignancy, CCR4-expressing Treg interacts with CCL17 and CCL22-secreting tumor cells, with resultant impairment of host antitumor immunity (31). Increase of CCR4 on the cell surface activates the PI3K/AKT signaling pathway to promote cell survival (26, 32). In this context, the anti-CCR4 monoclonal antibody mogamulizumab is already approved in Japan for ATLL treatment. However, there are concerns that mogamulizumab could induce adverse events, and that immunological statuses of patients may also affect treatment outcome (33). Therefore, alternative drug targets for ATLL are needed and are undergoing active investigation (**Supplementary Table 1**).

HTLV-1 Tax protein can infect and transform not only T-cells, but also various cell types including epithelial cells and fibroblasts (28, 34). In this regard, the microenvironment may contribute to survival and drug response of ATLL. Little is known about the role of stromal cells in ATLL, whereas in Hodgkin lymphoma (HL), it is suggested that the secretion of extracellular vesicles from HL changes the phenotype of fibroblasts to support tumor growth (35). In the case of T-cell lymphoma and leukemia, FGFR fusion genes are frequently found (36). During cancer progression, FGFR mediates crosstalk of CAFs with cancer cells and related target signaling pathways (37). Moreover, as a transmembrane growth factor, FGFR can activate the PI3K/AKT pathway that is closely related to ATLL (38).

In this study, we observed that the clonally expanded malignant tumor cells in ATLL are CD4 T-cells through scRNA-seq combined with TCR clonal analysis. We also identified the characteristics of CAFs within ATLL, including minimal expression of ACTA2 and PDGFRB, but high expression of FAP and PDGFRA. In particular, we identified a novel subgroup of CAFs characterized by high expression of EGR genes that may play an important role in the conditioning of the TME. We found that malignant T-cells and CAFs contribute to each other bidirectionally in ATLL, with CAFs promoting the clonal expansion of CD4 T-cells mediated by FGF7-FGFR1 signaling, and proliferating CD4 T-cells contributing to the growth of CAFs *via* PDGFA-PDGFRA/PDGFRB signaling.

## Data Availability Statement

The original contributions presented in the study are publicly available. This data can be found here: https://www.ncbi.nlm.nih.gov/geo/query/acc.cgi?acc=GSE195674.

## Ethics Statement

The studies involving human participants were reviewed and approved by IRB# SMC 2020-03-060. The patients/participants provided their written informed consent to participate in this study.

## Author Contributions

EHJ and JHB contributed equally. All authors contributed to the article and approved the submitted version.

## Funding

This research was supported by a grant of the Korea Health Technology R&D Project through the Korea Health Industry Development Institute (KHIDI), funded by the Ministry of Health & Welfare, Republic of Korea (grant number: HR20C0025) and by Basic Science Research Program through the National Research Foundation of Korea (NRF), funded by the Ministry of Education, Republic of Korea (grant number: 2020R1A6A1A03047972).

## Conflict of Interest

The authors declare that the research was conducted in the absence of any commercial or financial relationships that could be construed as a potential conflict of interest.

## Publisher’s Note

All claims expressed in this article are solely those of the authors and do not necessarily represent those of their affiliated organizations, or those of the publisher, the editors and the reviewers. Any product that may be evaluated in this article, or claim that may be made by its manufacturer, is not guaranteed or endorsed by the publisher.

## Supplementary Material

Supplementary Figure 1 Functional gene expression of T-cell subtypes.

Supplementary Figure 2 Differentially expressed gene (DEG) analysis of T-cells of ATLL compared to healthy donors **(A)** Volcano plot represents ATLL-specific DEGs. Cut-off of p-value < 0.05 and Log2 fold-change value > 1. **(B)** Gene ontology terms enriched in ATLL are highly related to tumor metabolic process. **(C)** Diagram of DEG analysis steps for clonal-specific gene expression within T-cells of ATLL.

Supplementary Figure 3 Characteristics of DC in ATLL **(A)** Correlation plot showing correlation coefficient between each myeloid cell type using variable feature genes (Spearman's rank correlation coefficient of TAM between DC = 0.51). **(B)** Expression level of inflammatory genes in DC.

Supplementary Figure 4 Network of FGFR-related tyrosine kinase inhibitor (TKI) target genes between ATLL marker genes.

Supplementary Table 1 Candidates of precision medicine.
