# Supplementary figures and images for: Deconvolution of Adult T-Cell Leukemia/Lymphoma With Single-Cell RNA-Seq Using Frozen Archived Skin Tissue Reveals New Subset of Cancer-Associated Fibroblast
Source: Front Immunol. 2022 Apr 7;13:856363. doi: 10.3389/fimmu.2022.856363 (PMC9021607; doi:10.3389/fimmu.2022.856363)

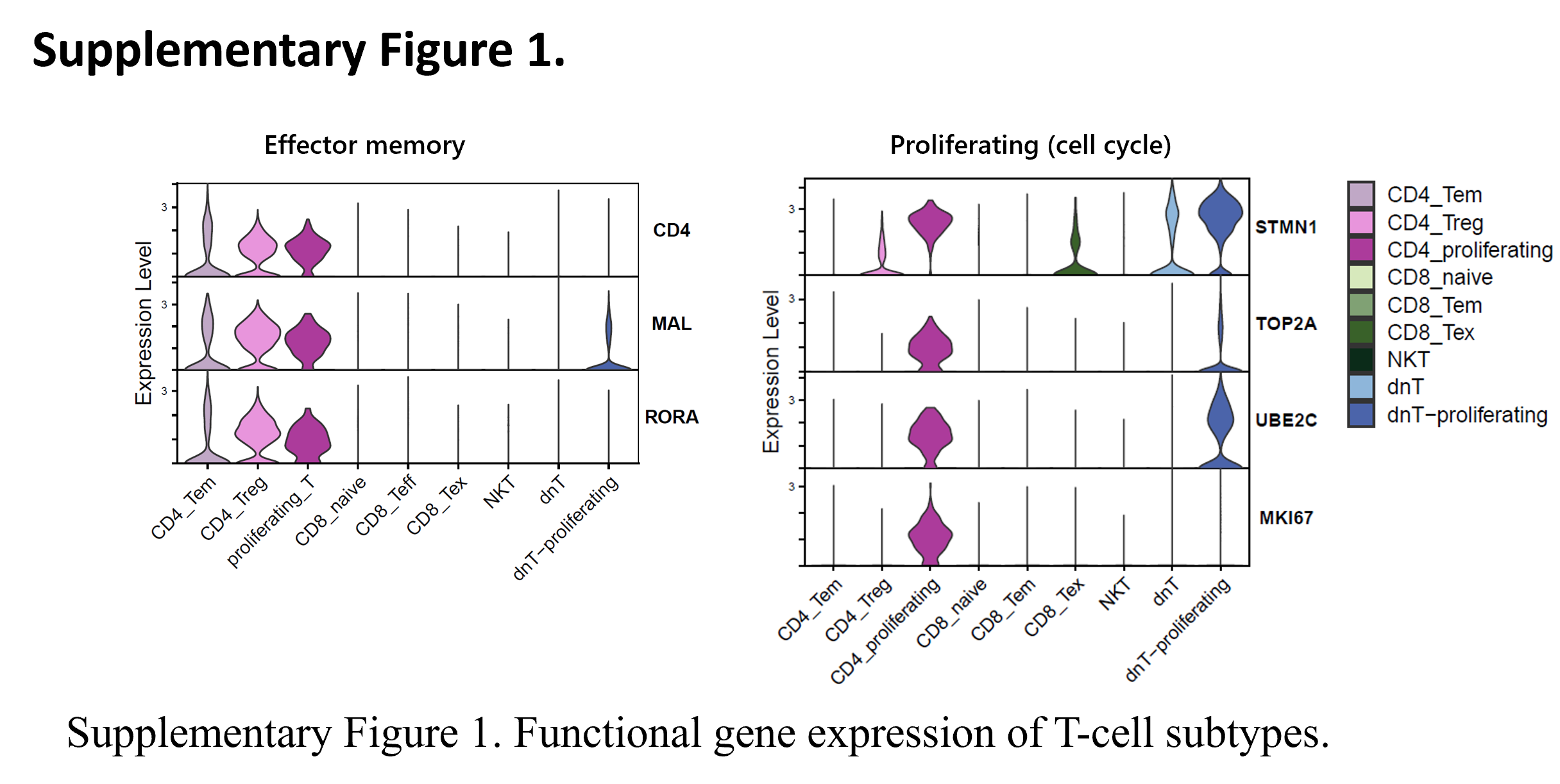

Supplement: Supplementary Figure 1 — Functional gene expression of T-cell subtypes. [file Image_1.tif]

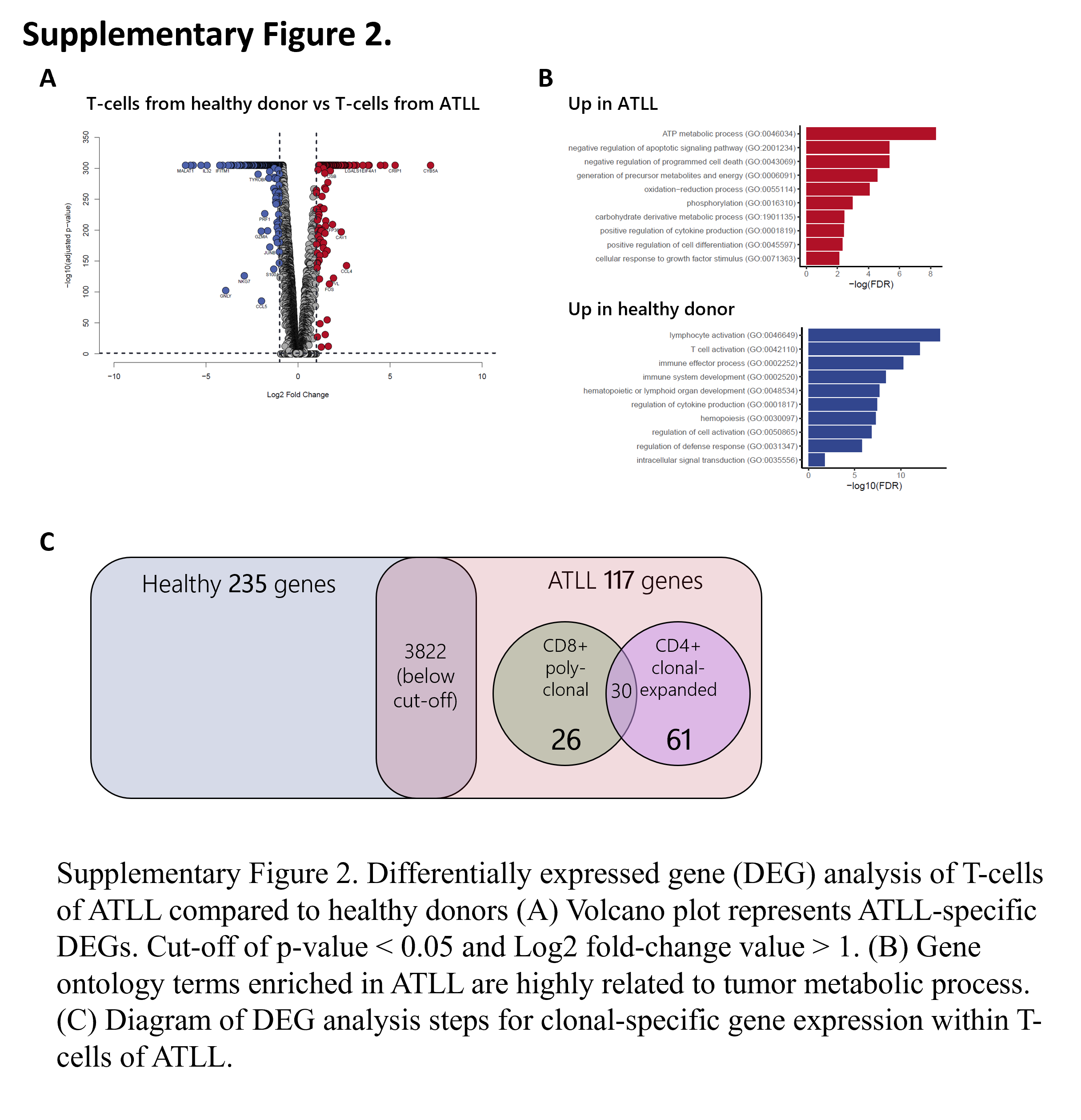

Supplement: Supplementary Figure 2 — Differentially expressed gene (DEG) analysis of T-cells of ATLL compared to healthy donors (A) Volcano plot represents ATLL-specific DEGs. Cut-off of p-value < 0.05 and Log2 fold-change value > 1. (B) Gene ontology terms enriched in ATLL are highly related to tumor metabolic process. (C) Diagram of DEG analysis steps for clonal-specific gene expression within T-cells of ATLL. [file Image_2.tif]

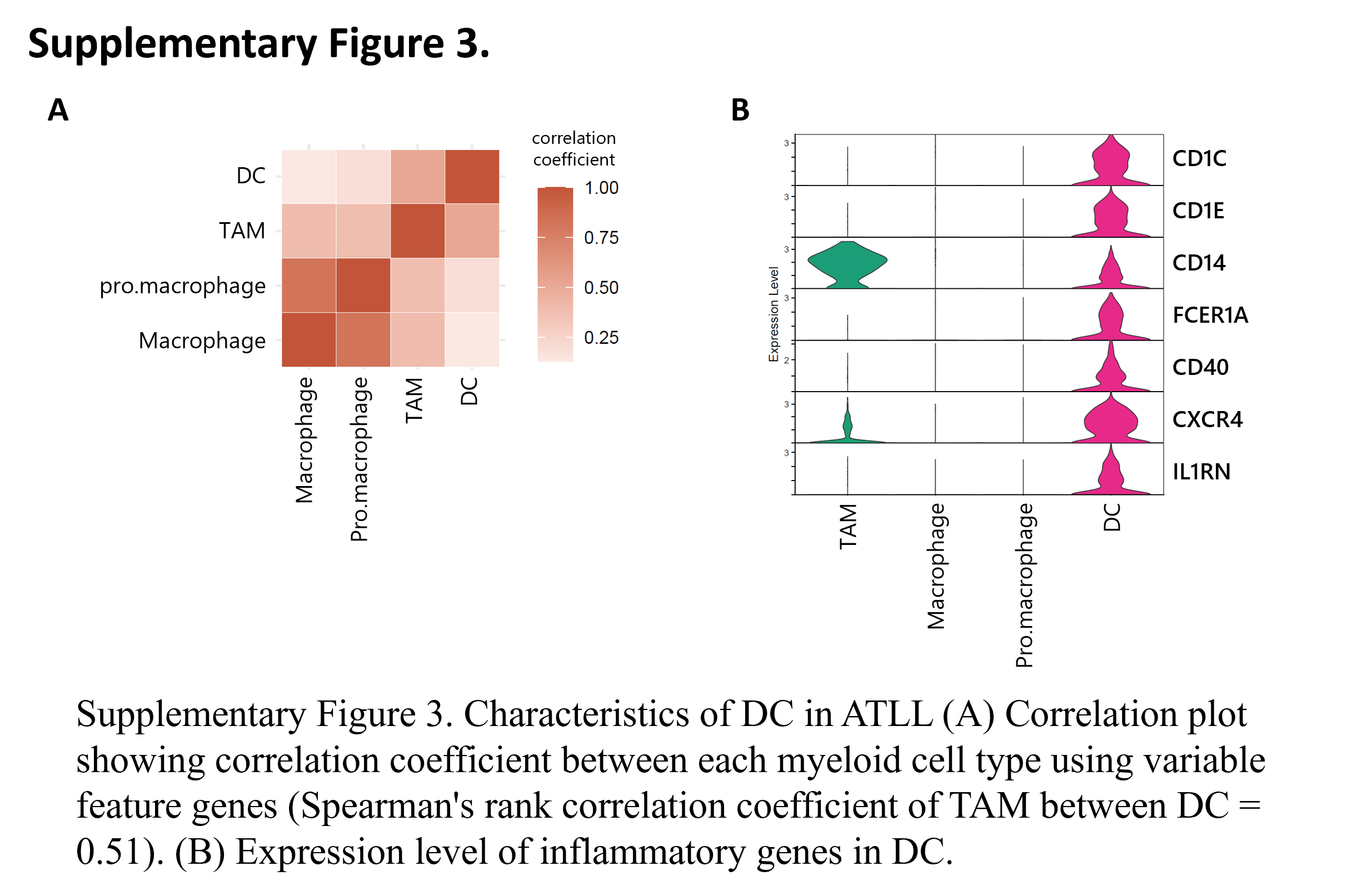

Supplement: Supplementary Figure 3 — Characteristics of DC in ATLL (A) Correlation plot showing correlation coefficient between each myeloid cell type using variable feature genes (Spearman's rank correlation coefficient of TAM between DC = 0.51). (B) Expression level of inflammatory genes in DC. [file Image_3.tif]

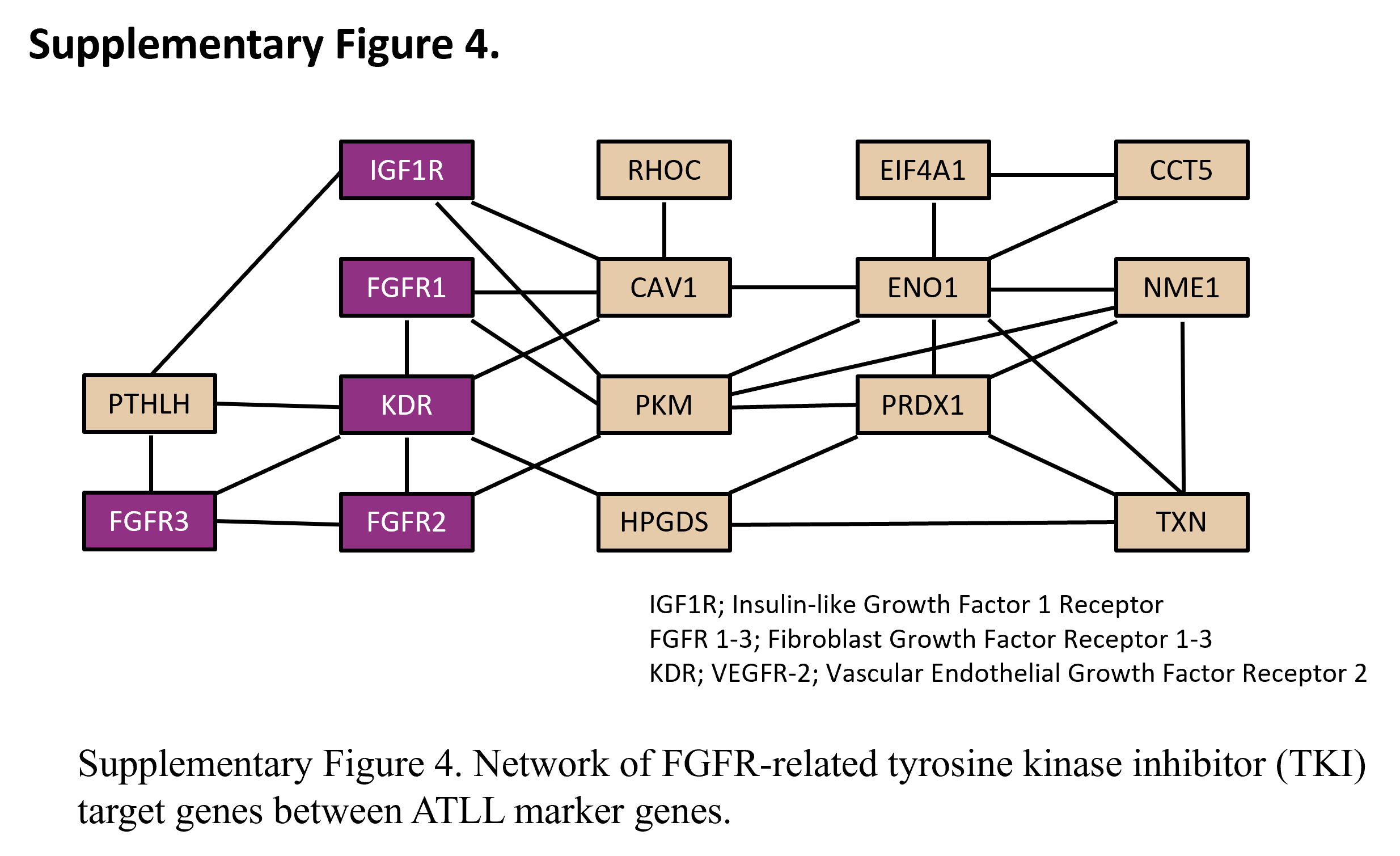

Supplement: Supplementary Figure 4 — Network of FGFR-related tyrosine kinase inhibitor (TKI) target genes between ATLL marker genes. [file Image_4.tif]
