# Supplementary material for: Deconvolution of Adult T-Cell Leukemia/Lymphoma With Single-Cell RNA-Seq Using Frozen Archived Skin Tissue Reveals New Subset of Cancer-Associated Fibroblast
Source: Front Immunol. 2022 Apr 7;13:856363. doi: 10.3389/fimmu.2022.856363 (PMC9021607; doi:10.3389/fimmu.2022.856363)
Supplement: Supplementary Table 1 — Candidates of precision medicine. [file Table_1.pdf]

Supplementary Table 1. Candidates of precision medicine

Target	Drug	Approval	Disease
FGFR1 (TKI target)	Ponatinib	Approved	Chronic myelogenous leukemia (CML) and acute lymphoblastic leukemia (ALL)
	Dovitinib	NDA submission	Renal cell carcinoma (RCC)
	AZD4547	In clinical studies	Lymphomas and myelomas
CCR4	Mogamulizumab	Approved	ATLL and CTCL
LAG3	IMP321(efti)	In clinical studies	NSCLC and HNSCC
	Relatlimab	In clinical studies	Melanoma
	GSK2831781	In clinical studies	Autoimmune diseases
CTLA4	Ipilimumab	Approved	Melanoma
	Tremelimumab	In clinical studies	Melanoma
CD27	Varlilumab	In clinical studies	Solid tumors and hematologic malignancies
IL2RA	Daclizumab (Zinbryta)	Approved	Immune-mediated disorders and breast cancer
